# UScale: a digital device for automatic urine volume measurement and frequency volume charting

**DOI:** 10.1177/1756287219875586

**Published:** 2019-09-19

**Authors:** James Williamson, Tahir Qayyum, Nicolas Bryan, Liam Blunt

**Affiliations:** Centre for Precision Technologies, University of Huddersfield, Haslett Building 3/07, West Yorkshire HD13DH, UK; Calderdale and Huddersfield NHS Foundation Trust, Huddersfield, UK; Calderdale and Huddersfield NHS Foundation Trust, Huddersfield, UK; Centre for Precision Technologies, University of Huddersfield, Huddersfield, UK

**Keywords:** bladder diary, frequency volume charts, UScale, voiding diary

## Abstract

**Background:**

Health issues relating to the lower urinary tract are an increasing burden on the health economy. Measurement of urination frequency/volume using diaries to evaluate symptoms and assess severity is established in the management of these health problems. In current practice, these frequency volume diaries are completed by voiding into a measuring jug and the completion of paper or digital charts. Despite being shown useful to diagnosis, this can be a cumbersome method of data collection, leading to issues with patient compliance. In this paper we describe the established benefits of providing clinicians accurate micturition data followed by an analysis of the problems with the current data collection method.

**Methods:**

We introduce our prototype electronic device and accompanying method, which is designed to improve data accuracy and patient compliance, while reducing patient training requirements and clinician workload.

**Results:**

The device hardware calibration and testing procedure is described, and two sets of initial data from assumed healthy volunteers are presented, allowing us to demonstrate the advantages of digital data in the fast calculation of diary summary statistics and their potential use to clinicians.

**Conclusions:**

We discuss the design improvements to the UScale device, collection bag, and electronic medical records integration undertaken while validating our described method.

## Introduction

It has long been suggested that lower urinary tract symptoms (LUTS) can be divided into voiding, storage, and postmicturition symptoms.^1^ These symptoms are highly prevalent,^2,3^ impairing quality of life (QoL),^4,5^ with an associated cost, and are likely to increase in the future.^6^ Taking a history is a fundamental process in the assessment of these symptoms, aiming to identify exacerbating and causative factors as well as comorbidities. The patient history should include a self-completed and clinically validated questionnaire alongside frequency/volume charts and bladder diaries.

Parameters derived from diaries include; day/night time voiding frequency, volume of individual voids, nocturnal volume, total voided volume, and mean voided volume. These data have been shown to be specifically useful for diagnosis in those with various LUTS.^7^ Variation in these parameters can be substantial, but the longer the diary duration, the less parameter variation^8,9^; however, multiple authors have suggested that diary study lengths of 3–7 days are sufficient to provide reliable micturition data.^10–12^

Colley states that patient compliance is an issue, as the accepted method of measuring and recording these parameters involves the cumbersome process of voiding into a jug/beaker and manually recording volumes.^13^ Furthermore, the use of a jug as a measurement vessel is not conducive to discrete and dignified use, discouraging continuation of normal routine in ambulant patients, leading to low compliance. Lastly, data accuracy using a jug is limited by patient training and the measurement vessel accuracy (±10%).^14,15^

In this paper, we describe a digital, gravimetric method of recording urination volume and time, thus facilitating automatic creation of micturition diaries. Our described method and prototype device are designed to operate with any fold-flat, hanging collection vessel, with the aim of providing patients with a device that is simple, intuitive, dignified, and discrete to use. This will allow continuation of normal patient routine, which we propose will increase patient compliance. The method and calibration is described before presenting initial results with two male volunteers, to demonstrate the availability of improved statistics for clinicians.

## Methodology

**Figure 1.**
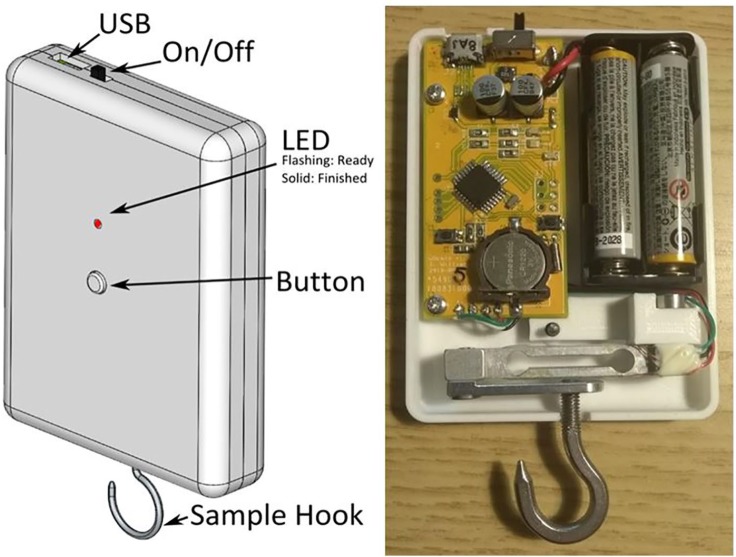
A CAD render of the device prototype (left) and an internal view of one of our first batch of prototype devices (right). CAD, computer-aided design.

At the start of the bladder diary study period, the patient is provided with a small (110 mm × 62 mm × 18 mm), light (80 g) and inexpensive (<£100) portable electronic device, UScale (Figure 1), and a supply of sample collection bags sufficient to last for the duration of the study period. Verbal training is provided by the care provider on the simple one-button operation of the UScale device and the method of sample collection, to be used for every micturition event during the study period.

When voiding, patients are asked to urinate into a modified low-cost polythene lined emesis bag. The bags are fold-flat making them discrete, and are single use, and, hence, hygienic compared with a jug. The wide bag openings make these bags usable by all. At this stage only men have used the bag, whilst future design work will aim to develop individualized shapes for men and women and study the change in compliance. After collection, the bag is able to stand upright, while the patient turns on the UScale device. The device then quickly zeroes the internal scale, after which an LED flashes to indicate readiness to measure.

The collected sample is hung from underneath the UScale device by an integral bag loop. A single button press is all that is required to perform the instantaneous measurement, after which the LED turns solid red to indicate that measurement is complete, the device can be turned off and the mass and time have been stored internally. After filling the bag, a full measurement cycle from turn-on to turn-off takes <10 s. Measuring inferred volume by mass removes the training requirements and inaccuracies of reading from graduated containers which are unsuitable for accurate volume measurement.

The contents of the bag can now be flushed and the bag disposed of with normal sanitary products, or sealed in a supplied bag for later discrete disposal. We believe the improved ease of use will increase patient uptake and conformance by facilitating a normal patient routine to continue throughout the study period, and we foresee the device being easy enough to use at work and while away from home, a major limitation of the current jug method.

This measurement process is then repeated for each micturition event until the end of the period of study, at which point the UScale device is returned to the clinician at the next clinical appointment. Retrieval of data is fast and easy, with connection of the device by USB, and download and analysis of data taking <10 s. Following transfer of the data to the clinician’s computer, key metrics are automatically calculated and are designed to reduce time from the study end to prognosis, reducing patient discomfort, staff overheads, and eliminating data entry inaccuracies.

**Figure 2.**
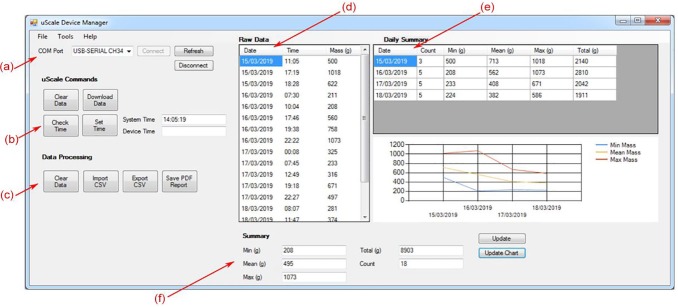
Screenshot of the prototype software interface for the UScale device working on a Windows 10 PC. Illustrates controls for device management, transfer of data, tables to view and edit raw data, and a summary of the bladder diary data. Labels (a)–(f) describe important functionality/features of the software: connection to device *via* USB interface (a); buttons to control the UScale device including setting the time, clearing data, and downloading data (b); buttons to calculate bladder diary summaries as well as import and export reports for saving to patient record (c); a summary of the raw data with functionality to edit or delete erroneous entries (d); Summary of each daily min, mean, max, and total volume and the frequency of micturition events (e); and an overall summary (f).

Figure 2 shows a screenshot of the UScale device management software for use on a personal computer by medical professionals. It is intended that this software will, in future, be a web-based interface to allow cross-platform compatibility, independent of medical trust hardware. The data in the screenshot above, and that in the methodology section, was collected by use of the UScale device by two of the authors using UScale for 3 days each. The labels (a)–(f) in Figure 2 describe important functionality/features of the software.

## Device calibration

This section details the process of calibrating and testing a UScale device, confirming parameters such as linearity, range, and resolution. These operations must be completed before a new device is released to the field, or intermittently to ensure device performance.

For calibration tests, a set of nine brass weights and a hanger acted as accurate mass simulators in place of urine to test the device from 0 to 1000 g in 100 g increments. Each of the brass masses were weighed using a precision balance (oHaus Galaxy 110), which the manufacturer informs has a stability of ±0.0001 g. The resulting values were used for calibration of the digital device, as well as to determine linearity, range, and resolution.

Each time the output from the UScale load cell is read by the microcontroller, we obtain a raw measurement, which is susceptible to time-varying electro-mechanical noise. To limit the effect of noise sources, such as shaking hands and moving samples, the mean of multiple raw measurements is used to calculate a measurement result, effectively filtering out most of the electro-mechanical noise.

Device calibration is the process of calculating the scaling factor required for the internal microcontroller to convert the signal from the force-sensitive load cell into a usable mass value. To calibrate the device, the scale is zeroed at start up, and a precision measured mass is then hung from the UScale. A result consisting of the mean of 50 raw measurements is divided by the known calibration mass to obtain the device scaling factor, which is programmed into the device software and allows UScale to accurately convert all subsequent results into grams.

The linearity of the device was calculated by adding masses to the device in 100 g increments and obtaining results at each increment. A least squares line of best fit was then calculated, and the maximum deviation from linear informed the linearity to be 99.94%. This signifies that the maximum deviation from linear across the whole range from 0 to 1000 g is ±0.59 g.

The repeatability of UScale was assessed across the 1000 g range by calculating the standard deviation (SD) of 100 results at each 100 g mass increment. To negate the effects of electrical and mechanical noise, each result was calculated as the mean of 20 raw measurements. The highest SD value was taken as the repeatability with a value of 1.4 g (1 SD). This value is a measure of the closeness of agreement between successive measurements, which, due to the environmental effects described above, is greater than the <0.1 g resolution of the device.

This section has reviewed the process undertaken to calibrate the UScale measurement device and to assess the quality of the measurement results.

## Results

In this section, we present the results from two bladder diaries recorded by the authors, assumed healthy volunteers using the prototype UScale device. In this paper, we make no attempt to diagnose symptoms or further prove the validity of the data for diagnosis, but we include these results to demonstrate the ability of the device to collect data, and to show that the use of digital data enables fast and clear summarization of recorded data.

For each bladder diary, the volunteers were provided with sufficient polythene lined paper bags and a UScale device each, and then completed a digital bladder diary for 3 full days. The summarized data can be seen in Tables 1 and 2, with both the daily and overall frequency, min, mean, max, and total mass calculated automatically by the software and provided to the clinician in an easy to understand table.

**Table 1.** Summary of bladder diary data for volunteer 1.

Date	Frequency	Min (g)	Mean (g)	Max (g)	Total (g)
15 March 2019	3	500	713	1018	2140
16 March 2019	5	208	562	1073	2810
17 March 2019	5	233	408	671	2042
18 March 2019	5	224	382	586	1911
Summary	18	208	516	1073	8903

**Table 2.** Summary of bladder diary data for volunteer 2.

Date	Frequency	Min (g)	Mean (g)	Max (g)	Total (g)
20 March 2019	2	369	405	441	810
21 March 2019	6	71	241	388	1448
22 March 2019	8	82	311	624	2488
23 March 2019	5	97	187	268	935
Summary	21	71	286	624	5681

This section has demonstrated the ability of UScale to automatically measure the date, time, and mass of micturition events over a 3-day bladder diary period on a single battery charge, and provide a fast, automatically calculated, summary to the clinician. This level of data analysis was not previously possible with paper-based charting without time consuming input and analysis by medical staff.

In the future, these mass values will be multiplied by a scaling factor dictated by the specific gravity of urine (1.001–1.030), providing a volume result in milliliters. The specific gravity scaling factor will be chosen to minimize the error introduced by differing urine densities, the implications of which are discussed in the following.

## Discussion

Having assessed numerous parameters of the UScale device, and demonstrated the ability to make field measurements, the authors feel that, following ethical and regulatory advice, this method is ready for small-scale tests with patients and medical staff. This will seek to assess change in patient compliance and gain feedback on the practical aspects of the methodology from patients and clinicians alike, informing us of patient and staff training requirements, acceptance of the method by patients, sample bag, and device ergonomics, as well as allowing field testing of the device. We consider the ease of this method, due to single button operation and reduced training requirements, to be a significant improvement over paper-based charting for patients.

The digital nature of the data will result in time savings for clinicians, as the data will be available and summarized instantly, with the ability to later compare anonymized data with demographics and medication factors, representing a potential valuable tool to research communities. Meanwhile, we intend to make hardware and software design changes to provide our device with the ability to wirelessly upload data to a secure online database upon return to the clinician. This will remove the requirement for installation on healthcare provider systems, providing access to the summarized data *via* an easy-to-use web browser interface. This is intended to further reduce the workload for medical staff and will, in future, facilitate automatic integration with patient electronic medical records.

The conversion from mass to volume must also be considered. Utilizing a gravimetric method of assessment of voided volume is a common feature found in urine flow machines^16,17^; however, these devices are not portable and measure continuous urine production. A consideration for gravimetric assessment of urine volume is the effect of urine specific gravity (USG), the measurement of the concentration of particles in a solution. The specific gravity of water and urine is 1.000 and 1.001–1.035, respectively, with the specific gravity of urine varying with factors such as hydration, presence of glucose, and protein, among others. Given the normal variation of USG, and this having limited clinical impact on flow parameters when assessed by gravitational flow machines, we feel this should not affect the utility of the UScale device. In future work, we propose to choose a representative average USG to improve the accuracy of converting from mass to volume, or, alternatively, to provide clinicians with a volume error range, calculated automatically using typical USG values.

We also foresee the addition of an inexpensive wrist-worn fitness tracker to our method. While our current method stores time and date of urination events, it does not inform whether an event is during a sleep cycle, thus losing the direct assessment of nocturnal frequency/volume and total nocturnal volume. We propose that the inclusion of a commercially available fitness tracker will allow us to correlate urination times to sleep interruption, voiding patterns, and volumes, which cause significant morbidity to patients.^18^

Finally, while making these improvements to the device and method, we plan to involve patient and clinician groups to prove conclusively whether the use of a simple technological aid such as UScale provides value to patients and clinicians over existing manual bladder diary methods. This will lead to formal assessment of different groups to define what is ‘normal’ and, subsequently, a comparison with patient groups.

## Conclusion

This paper has reported a method for automated bladder diary measurement as well as the development, calibration, and usage of hardware and software to facilitate and support this new method in the form of a prototype device, UScale. We propose that this device will improve the comfort, confidence, and conformance of patients completing bladder diaries, while reducing the cost associated with such tests and improving their value to medical professionals. Lastly, we propose changes to the device designed to further increase the advantages to patients and clinicians, as well as describe future work to assess these claims in a clinical setting.

## References

[1] Abrams P Cardozo L Fall M, et al The standardisation of terminology in lower urinary tract function: report from the standardisation sub-committee of the International Continence Society. Urology 2003; 61: 37–49. 12559262 10.1016/s0090-4295(02)02243-4

[2] Britton JP Dowell AC Whelan P. Prevalence of urinary symptoms in men aged over 60. Br J Urol 1990; 66: 175–176. 2390704 10.1111/j.1464-410x.1990.tb14898.x

[3] van Dijk L Kooij DG Schellevis FG Nocturia in the Dutch adult population. BJU Int 2002; 90: 644–648. 12410740 10.1046/j.1464-410x.2002.03011.x

[4] Martin SA Haren MT Marshall VR, et al Prevalence and factors associated with uncomplicated storage and voiding lower urinary tract symptoms in community-dwelling Australian men. World J Urol 2011; 29: 179–184. 20963421 10.1007/s00345-010-0605-8

[5] Agarwal A Eryuzlu LN Cartwright R, et al What is the most bothersome lower urinary tract symptom? Individual-and population-level perspectives for both men and women. Eur Urol 2014; 65: 1211–1217. 24486308 10.1016/j.eururo.2014.01.019 PMC4018666

[6] Taub DA Wei JT. The economics of benign prostatic hyperplasia and lower urinary tract symptoms in the United States. Curr Urol Rep 2006; 7: 272–281. 16930498 10.1007/s11934-996-0006-0

[7] National Institute for Health and Care Excellence. Lower urinary tract symptoms in men: management, https://www.nice.org.uk/guidance/cg97/chapter/1-Recommendations#initial-assessment-2 (2010, accessed 10 June 2019). 31999413

[8] Konstantinidis C Kratiras Z Samarinas M, et al Optimal bladder diary duration for patients with suprapontine neurogenic lower urinary tract dysfunction. Int Braz J Urol 2016; 42: 766–772. 27564288 10.1590/S1677-5538.IBJU.2015.0292 PMC5006773

[9] Homma Y Ando T Yoshida M, et al Voiding and incontinence frequencies: variability of diary data and required diary length. Neurourol Urodyn 2002; 21: 204–209. 11948713 10.1002/nau.10016

[10] Abrams P Klevmark B. Frequency volume charts: an indispensable part of lower urinary tract assessment. Scand J Urol Nephrol Suppl 1996; 179: 47–53. 8908664

[11] Dmochowski RR Sanders SW Appell RA, et al Bladder-health diaries: an assessment of 3-day vs 7-day entries. BJU Int 2005; 96: 1049–1054. 16225527 10.1111/j.1464-410X.2005.05785.x

[12] Wyman JF Choi SC Harkins SW, et al The urinary diary in evaluation of incontinent women: a test-retest analysis. Obstet Gynecol 1988; 71(6 Pt 1): 812–817. 3368165

[13] Colley W. Use of frequency volume charts and voiding diaries. Nurs Times 2015; 111: 12–15.

[14] International Organisation for Standardization. BS ISO 3819:1985 Laboratory glassware – beakers. BSI Standards Publication 1985.

[15] International Organisation for Standardization. ISO 7056: 1981 Plastics laboratory ware – beakers. BSI Standards Publication 1981.

[16] Otero A Palacios F Akinfiev T, et al A device for automatically measuring and supervising the critical care patient’s urine output. Sensors (Basel) 2010; 10: 934–951. 22315578 10.3390/s100100934 PMC3270879

[17] Chang AJ Nomura Y Barodka VM, et al Validation of a real-time minute-to-minute urine output monitor and the feasibility of its clinical use for patients undergoing cardiac surgery. Anesth Analg 2017; 125: 1883–1886. 29190218 10.1213/ANE.0000000000002217 PMC5726301

[18] van Doorn B Kok ET Blanker MH, et al Mortality in older men with nocturia. A 15-year followup of the Krimpen study. J Urol 2012; 187: 1727–1731. 22425119 10.1016/j.juro.2011.12.078

